# How Attention Modulates Encoding of Dynamic Stimuli

**DOI:** 10.3389/fnhum.2016.00507

**Published:** 2016-10-21

**Authors:** Noga Oren, Irit Shapira-Lichter, Yulia Lerner, Ricardo Tarrasch, Talma Hendler, Nir Giladi, Elissa L. Ash

**Affiliations:** ^1^Sackler Faculty of Medicine, Tel Aviv UniversityTel Aviv, Israel; ^2^Tel Aviv Center for Brain Functions, Tel Aviv Sourasky Medical CenterTel Aviv, Israel; ^3^Department of Neurology, Tel Aviv Sourasky Medical CenterTel Aviv, Israel; ^4^Sagol School of Neuroscience, Tel Aviv UniversityTel Aviv, Israel; ^5^School of Education, Tel Aviv UniversityTel Aviv, Israel; ^6^School of Psychological Sciences, Tel Aviv UniversityTel Aviv, Israel; ^7^Center for Memory and Attention Disorders, Tel Aviv Sourasky Medical CenterTel Aviv, Israel

**Keywords:** episodic memory, intersubject correlation, divided attention, dual task, fMRI, movie, posterior cingulate cortex, ventro lateral prefrontal cortex

## Abstract

When encoding a real-life, continuous stimulus, the same neural circuits support processing and integration of prior as well as new incoming information. This ongoing interplay is modulated by attention, and is evident in regions such as the prefrontal cortex section of the task positive network (TPN), and in the posterior cingulate cortex (PCC), a hub of the default mode network (DMN). Yet the exact nature of such modulation is still unclear. To investigate this issue, we utilized an fMRI task that employed movies as the encoded stimuli and manipulated attentional load via an easy or hard secondary task that was performed simultaneously with encoding. Results showed increased intersubject correlation (inter-SC) levels when encoding movies in a condition of high, as compared to low attentional load. This was evident in bilateral ventrolateral and dorsomedial prefrontal cortices and the dorsal PCC (dPCC). These regions became more attuned to the combination of the movie and the secondary task as the attentional demand of the latter increased. Activation analyses revealed that at higher load the prefrontal TPN regions were more activated, whereas the dPCC was more deactivated. Attentional load also influenced connectivity within and between the networks. At high load the dPCC was anti-correlated to the prefrontal regions, which were more functionally coherent amongst themselves. Finally and critically, greater inter-SC in the dPCC at high load during encoding predicted lower memory strength when that information was retrieved. This association between inter-SC levels and memory strength suggest that as attentional demands increased, the dPCC was more attuned to the secondary task at the expense of the encoded stimulus, thus weakening memory for the encoded stimulus. Together, our findings show that attentional load modulated the function of core TPN and DMN regions. Furthermore, the observed relationship between memory strength and the modulation of the dPCC points to this region as a key area involved in the manipulation of attentional load on memory function.

## Introduction

Traditional theories postulate that the information required for current task performance is actively maintained in a designated working memory component (Baddeley, 2003). This view dissociates between processing and activating information. Contemporary views, on the other hand, see these two operations intertwined (Fuster, 1997; Hasson et al., 2015). Combining the latter idea with recent imaging findings, Hasson et al. (2015) have outlined how the same neural circuits support processing of both new incoming and preceding information when facing continuous stimuli that approach real-life, such as movies or narrated stories. In this framework, the interplay between information processing and traces of prior events can be modulated by attention. While this framework is based on a wide-range of experimental findings with continuous stimuli, the notion that memory is modulated by attention has been demonstrated only with still stimuli. Here we used functional magnetic resonance imaging (fMRI) to study how attention modulates the encoding of continuous stimuli, while systematically changing the amount of attentional resources available during encoding. Movies were employed as the encoded stimuli and inter-SC was used as the key neural measure.

The framework of Hasson et al. (2015) postulates that when facing a real-life, continuous stimulus, the constantly incoming new input is integrated with the information that preceded it. This processing-memory interplay is best captured in the lab when displaying stimuli that approach real-life, such as movies, and analyzing the moment-by-moment neural dynamics across participants, i.e., the reliability of evoked neural activity, or synchronization, quantified as inter-subject correlation (inter-SC; Hasson et al., 2004, 2010; for further details regarding inter-SC see the Materials and Methods section). Studies using this method have shown that the integration happens in almost all cortical circuits, yet regions differ from one another in the timescale of this process (Hasson et al., 2015). Primary sensory cortices accumulate only several milliseconds of information, meaning that only a few milliseconds or seconds of the past are maintained activated during stimulus processing (Hasson et al., 2008b; Lerner et al., 2011). In contrast, higher order areas, including the ventrolateral and dorsomedial prefrontal regions, as well as the posterior cingulate cortex (PCC), can integrate information across many seconds and minutes (Lerner et al., 2011). Thus, at the end of a scene which lasts a few minutes, these areas still hold traces of the beginning of the scene active (reviewed in Hasson et al., 2015).

However, using active traces of past information to process current information does not happen in isolation, but rather is modulated by attention (Fougnie, 2008). Numerous studies that utilized still stimuli have shown that attending to a stimulus results in a higher memory strength for that stimulus and increased activation of regions such as prefrontal and parietal cortex and the hippocampi (Lepsien et al., 2011; Aly and Turk-Browne, 2016). Yet it is still unclear what would be the nature of such modulation under the assumption that the same circuits are involved in processing and holding the information active. The framework of Hasson et al. (2015) envisages that attentional modulation should mainly affect higher order regions, such as the PCC, that are known to accumulate information for more prolonged periods. Here we examined this prediction by manipulating attentional load via an easy or hard secondary task performed simultaneously while encoding a movie. We hypothesized that with increased attentional demand, the evoked neural activity of different people will become more similar since they have to pay greater attention to the ongoing stimuli. This would result in a higher level of inter-SC.

Attention is assumed to influence regions that are attributed to distinct neural networks, namely the prefrontal sections of the task positive network (TPN; Fox et al., 2005), and the PCC, which is the posterior hub of the default mode network (DMN; Raichle et al., 2001). This distinction is important since our hypothesis assumes that attentional load would be associated with higher inter-SC level in both networks, whereas attentional load may have opposite effects on these networks, specifically increased activation in the TPN and deactivation in the DMN. Therefore the inter-SC analysis was complemented by a standard activation analysis to distinguish TPN and DMN nodes. Furthermore, the DMN and TPN networks are known to be anti-correlated with one another (Fox et al., 2005), and particularly, the PCC is known to be both negatively correlated with prefrontal regions and to modulate their activity (Uddin et al., 2009). Using still stimuli, it has been shown that higher attentional load is associated with greater functional connectivity (FC) within the TPN and DMN and greater anti-correlation between the two (Newton et al., 2011). We assumed that the same patterns would be evident with continuous stimuli. To verify this assumption we examined FC within and between the networks at encoding as a function of load.

## Materials and Methods

### Participants

Twenty-four healthy adults completed the task [mean age (SD) 29 (3.7); mean education (SD) 15.37 (2.24); 8 females]. All participants were right-handed, except for two ambidextrous individuals with a tendency to use the right hand more, as indicated by the Hebrew translation of the Edinburgh Handedness Inventory (Oldfield, 1971). All participants were native Hebrew speakers. None of the participants had a history of neurological, psychiatric, or speech-related problems, and all had normal or corrected to normal vision. The fMRI data of two participants were discarded since they completed a version of the fMRI task which had shorter fixation periods. Note, however, that this difference in length of fixation was not anticipated to affect the behavioral response. The experimental procedures were approved by the Tel Aviv Sourasky Medical Center Institutional Review Board. Participants provided written informed consent.

### Stimuli and Experimental Design

#### Overview

**FIGURE 1:**
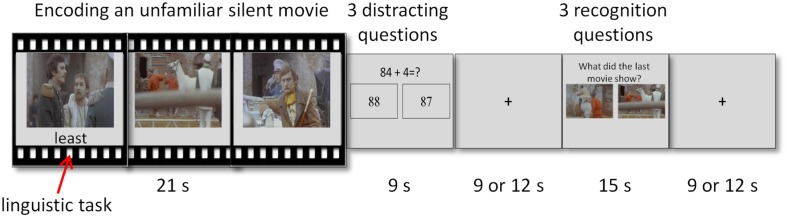
**Illustration of the experimental paradigm.** A cycle of the experimental paradigm composed of three phases – encoding, distraction, and recognition. Attention load was modified via an easy or hard secondary linguistic task performed during encoding.

The fMRI task examined episodic memory and consisted of a cycle of three phases – encoding, distraction, and recognition (**Figure 1**). A short movie was played during the encoding phase. The distraction phase immediately followed and consisted of mathematical questions meant to diminish rehearsal and recency effects (Baddeley, 2003). The third phase consisted of mnemonic recognition questions. A fixation cross of interchangeable length of 9 or 12 s was presented between the distraction and recognition phases, and between the recognition phase and the next cycle. Interchangeable length was used in order to minimize expectation effects. The 3-phase cycle repeated 24 times with different movies and questions in each cycle.

Two variables were manipulated during the encoding phase: attentional load and context. Changing attention load was done via an easy or a hard secondary linguistic task performed during encoding. Context was introduced by presenting 12 pairs of related movies in consecutive cycles. Attentional load and context manipulations were fully crossed. Thus, the experimental paradigm was a 2 × 2 design, with the following four conditions: low load-first movie, low load-second movie, high load-first movie, high load-second movie. Each condition was repeated six times, with the order of the combinations and runs randomized across participants. The current study focuses solely on attentional load and the context manipulation was thus irrelevant to the analyses. Therefore all analyses examined the difference between low and high attentional load, averaged across the first and the second movies (see below).

The paradigm was divided into three runs of 582 s each. A run started with a fixation cross for 24 s and a movie that was not followed by distraction or recognition phases. The purpose of this single movie was to habituate the neural response to the visual stimuli and to accustom the participants to the task. This movie was discarded from the analyses. A run consisted of 8 cycles of encoding, distraction and recognition phases. Participants responded to the linguistic, mathematical, and mnemonic tasks by pressing either the right or left button on a hand-held response box. Prior to the experiment, each participant received an explanation about the paradigm and practiced until he or she indicated that the task was clear. The practice session presented different stimuli from those used in the actual experiment.

#### Encoding Phase

During the encoding phase, a short, 21 s movie was presented. The movies in the task were unfamiliar, non-emotional, silent clips edited from feature length films. The original feature length films were not silent but sound was omitted during editing.

##### Context manipulation during the encoding phase

Context was achieved during encoding by presenting pairs of related movie clips in successive cycles. Each pair of movies portrayed the same characters in a similar setting performing a similar action. The resemblance between the clips in a pair of related movies was strong. The resemblance between unrelated pairs of movies was weak. Overall, there were 12 pairs of short related movies. The order of the movie clips within a pair (i.e., which movie was presented first and which second) was counter balanced across participants. Notably, each movie clip had its own recognition questions, independent of its paired movie clip. We remind the reader that for the purposes of this article, the context manipulation was irrelevant to the analyses and did not affect the attentional load manipulation (see below).

##### Attentional load manipulation during the encoding phase

Attentional load was changed during encoding by the simultaneous performance of an easy or a hard linguistic task. The task required participants to indicate whether a string of letters was a word or pseudo-word. Participants were requested to attentively watch the movie and perform the linguistic task as accurately as possible. Stimuli for the linguistic task were selected based on an independent behavioral pilot study conducted with a different group of participants (*n* = 19; mean age 33.4; 14 females) who performed this task alone. Accuracy and reaction time (RT) of 213 words and 197 pseudo-words were evaluated. The 54 stimuli with the shortest RT and highest accuracy level were selected for the easy category. The 54 stimuli with the longest RT and lowest accuracy level were selected for the hard category. In each category, half of the stimuli were words and half were pseudo-words. The easy and hard categories differed in terms of accuracy level and RT, as indicated by two *t*-tests [*t*_(18)_ = 10.76, *p* < 0.001; *t*_(18)_ = 10.11, *p* < 0.001; for accuracy level and RT, respectively]. Three words were presented during each movie: two words and one pseudo-word or vice versa. The words appeared one at a time, like subtitles below the movie, for 2 s each. Each word appeared 0.5 s before the frame that served as a target in the recognition questions. Consequently, for each movie the words were presented at the same exact time for all participants, regardless of the task type (low or high attentional load) and regardless of the order of the movie (first or second in a pair). The word disappeared when the participant pressed the response button or after the 2 s time frame elapsed. Overall, there were 12 movie clips (i.e., six pairs of movies) coupled with an easy linguistic task and 12 movie clips with a hard linguistic task (i.e., low and high attentional loads, respectively). The coupling of movie pairs to easy or hard linguistic task was counter balanced across participants.

#### Distraction Phase

During the distraction phase, three mathematical questions were presented for 3 s each. A question consisted of a simple equation (e.g., “56+7=?”) at the top of the screen, with the correct answer and a foil underneath it in two separate rectangles. If the response was given within the question’s time frame, the equation disappeared and the rectangles became blank.

#### Recognition Phase

During the recognition phase, three recognition questions were presented for 5 s each. The recognition questions were designed analogously to the mathematical questions. Namely, the question “What did the last movie show?” was presented at the top of the screen with two picture choices presented below. The correct answer was a picture frame taken from the short movie that immediately preceded the question. The foil was a frame taken from the original feature length film presenting the same characters and setting, but from a segment that did not appear in either of the two short movies viewed. If the response was given within the question’s time frame, the question disappeared and the rectangles became blank.

### MRI Acquisition

MRI scans were performed on a 3.0 Tesla MRI scanner (GE Signa EXCITE, Milwaukee, WI, USA) using an eight channel head coil. Blood-oxygen-dependent-level (BOLD) functional MRI was acquired with T2^∗^-weighted imaging: repetition time (TR) = 3000 ms; echo time (TE) = 35 ms; flip angle (FA) = 90°; field of view (FOV) = 200 mm; matrix size = 96 × 96; 39 axial slices of 3 mm thickness, 0 gap. A high-resolution anatomical T1-weighted fast spoiled gradient echo imaging was acquired: FOV = 256 mm; matrix = 256 × 256; TR = 9.2 ms; TE = 3.5 ms; axial slices of 1 mm thickness, no gap. This anatomical scan was used for surface reconstruction. To minimize head movements, participants’ heads were stabilized with foam padding. Stimuli were controlled using PsychoPy software (Peirce, 2007, 2008) and presented via an LCD projector to a tilted (45°) mirror positioned over participants’ foreheads. A MR-compatible response box was used to collect responses.

### Data Analysis

#### Behavioral Analysis

There were three behavioral tasks: linguistic (performed during the encoding phase), mathematical (performed during the distraction phase) and mnemonic (performed during the recognition phase). In each, accuracy level and mean RT were measured. The accuracy level was calculated as the proportion of correct responses. Questions with RT shorter than 0.1 s were discarded from analysis. Incorrect questions were discarded from the RT analysis.

The analyses commenced with validation of the distracting and attentional load manipulations. Analysis of the distracting mathematical task aimed to verify that the participants performed the task above chance level. To this end, accuracy level across all the experimental conditions was evaluated. The linguistic task was analyzed in order to confirm that it had indeed two difficulty levels. This was done using a paired-sample *t*-test, comparing low vs. high attentional loads. This was done once for accuracy level and once for RT.

Before analyzing the recognition questions, the efficiency of each question was examined using the data of an independent behavioral study with a different group of participants (*n* = 18; mean age 29.16; 9 females). These participants completed the same movies task (however, with shorter fixation interval of 3 or 4 s), while sitting in front of a laptop in a quiet room. Accuracy levels for each question were calculated across all participants. Three of the 72 questions had accuracy levels lower than 0.5 overall (i.e., across all the experimental conditions) and in the easiest condition (i.e., low load first movie), and were discarded from further analyses.

Following the aforementioned procedure, the recognition task was analyzed using a 2-way repeated measure analysis of variance (ANOVA) with context (first/second movie) and attentional load (low/high) as within-subject variables. There was neither effect for context nor any interaction between context and attentional load, for either accuracy level or RT (*p* > 0.05 for all). Therefore, the first and the second movies were collapsed together in all subsequent analyses.

#### fMRI Analysis

As elaborated above, in the current study only the effect of attentional load was analyzed by comparing the low and high attentional load conditions. fMRI data analysis was confined to the signal recorded during the encoding phase. Data were analyzed using the BrainVoyager QX software (Formisano et al., 2006; Goebel et al., 2006) and in-house codes implemented in MATLAB.

##### Preprocessing

Preprocessing of the functional scans included cropping of the first 6 TRs in each run to allow the hemodynamic responses to reach steady-state, 3D motion and slice scan time correction, linear trend removal and high-pass filtering (1 cycle per run). Head movements did not exceed 3 mm in any of the runs. Spatial smoothing was applied using a Gaussian spatial filter (6 mm full-width at half-maximum value). The functional images were superimposed on 2D anatomical images and incorporated into the 3D datasets through trilinear interpolation. The complete functional dataset was transformed into Talairach space (Talairach and Tournoux, 1988).

##### Inter-SC analysis

Inter-SC analysis measures the progressive changes in neural signal that arise when encountering dynamic naturalistic stimuli (Hasson et al., 2004, 2010). Here we were interested in identifying regions which changed their inter-SC level in response to the encoded movie as a function of attentional load. To this end, the following steps were performed. First, for every participant and movie, a Pearson correlation map was constructed by calculating, on a voxel-by-voxel basis (in Talairach space), the correlation between the BOLD signal of that participant and the averaged BOLD signal of all the other participants sharing the same experimental condition (e.g., between participant *k* and all the other participants who saw the same movie as a low load first movie). The averaged BOLD signal was constructed across 4 to 5 participants, depending on the movie. The correlation coefficients were transformed using a Fisher transformation. Note that in the inter-SC analytic method, the average time course of the participants serves as a model for the time course of the “left out” participant, and therefore, that model should include only participants which are identical to the participant with regard to the specific experimental condition. However, once the correlation maps were constructed, they can be averaged across conditions to create the contrast of interest, namely low vs. high attentional load. Since participants watched 12 movies within each manipulation condition (i.e., 12 low load and 12 high load movies), every participant had 12 correlation maps per experimental condition, which were averaged together, yielding one correlation map per condition for each participant.

Second, voxels in which the correlation coefficient was significantly higher than zero were identified. This stage prevented comparison of two non-significant correlations, which may be significant by itself, but bears no substantial meaning. For this, in every voxel, a one sample *t*-test was used cross all the 22 participants to determine whether the correlation was different from zero. This was done once for the low and once for the high attentional load conditions. Controlling for multiple comparisons across the whole brain was done using a false discovery rate (FDR) method (Benjamini and Hochberg, 1995; Benjamini and Yekutieli, 2001; Genovese et al., 2002), with a threshold of *p* < 0.05. The next stage of analysis was conducted only on voxels whose average correlation coefficient was significantly larger than zero in at least one condition.

Finally, we were interested to find voxels that had a significantly higher correlation in either low or high attentional load. Therefore, in every voxel, the difference between the correlation of the low and high attentional load conditions was examined across all 22 participants using a paired-sample *t*-test. For this *t*-test, controlling for multiple comparisons was done using the aforementioned FDR method, with a threshold of *p* < 0.05 and minimum cluster size of 50 × 3^3^ voxels. The results of the analysis are presented in a correlation map for each condition (averaged across the 22 participants; see Supplementary Figure S1) and in a t-map of the significant differences between the conditions (see Results section).

##### Characterizing signal level via activation analysis

We predicted that high attentional load would be associated with a higher level of inter-SC. However, a higher inter-SC level indicates only that the neural signal of a region became more correlated across participants, but not if the region was more activated or deactivated. In order to further characterize the level of the signal and sort the regions into TPN and DMN, the inter-SC analysis was complemented by a standard activation analysis. Activity level was examined in regions which demonstrated significant changes in inter-SC patterns using the following steps in each region. First, the timecourse of the encoding phase, averaged across all the voxels in a region, was extracted, incorporating a hemodynamic delay of 2 TRs (6 s). The timecourse was used to calculate the region’s percent signal change (%SC) from baseline in the low and high attentional load conditions. Second, across all participants, a one-sample *t*-test was used to determine whether the activation level in either the low or high attentional load condition was significantly different than zero (i.e., from baseline). Like in the inter-SC analysis, this step prevented comparison of two non-significant activations. Therefore, the next stage of analysis was conducted only in regions whose average activation was significantly larger than zero in at least one condition. Third, the difference between the low and high attentional load was determined using a paired-sample *t*-test. Regions were further sorted into TPN (%SC > baseline and high load > low load) or DMN (%SC < baseline and low load > high load). Only regions that fulfilled either of these criteria were regarded as regions of interest (ROI) and included in subsequent analyses.

##### Functional connectivity analysis

We examined the hypothesis that higher attentional load would be associated with greater FC within each functional network (i.e., TPN and DMN) and larger negative correlation between the networks. The analysis was conducted as follows for every possible pair of ROIs. First, for a given participant, the time course of each movie was extracted from a pair of ROIs, and a Pearson correlation was calculated between the two time courses. This resulted with 24 correlation coefficients for each participant, one for each movie, and all of them were Fisher-transformed. Second, all the correlations corresponding to the low attentional load condition were averaged together, and the same was done for the high attentional load condition. Third, across all participants, a one-sample *t*-test was used to determine whether the correlation in either the low or the high attentional load condition was significantly different than zero. The fourth step was conducted only in cases where at least one condition was significantly different than zero and consisted of a paired-sample *t*-test to assess the difference between the low and high attentional load conditions. Controlling for multiple comparisons was done using the Bonferroni method, taking into account the total number of paired comparisons.

##### Functional significance of inter-SC patterns

The final stage of analysis aimed to clarify the role of the identified inter-SC patterns in memory when dealing with increased attentional loading. To this end, a step-wise regression analysis was used. One model was built to predict accuracy level in the recognition task and another to predict RT. The predicting variables were the inter-SC patterns of all ROIs. Accuracy level, RT and inter-SC were modeled as the difference between high and low attentional load conditions (high load – low load; to be termed index). In order to verify that this brain-behavior relationship was specific to inter-SC, parallel models were calculated, once when the predicting variables were the ROIs’ activation indices and once when they were the FC indices.

## Results

### Behavioral Results

First, we validated the efficiency of the mathematical and linguistic tasks. In the former, the accuracy level was above chance [mean (SD): 88.7% (8.15)]. This indicates that participants were engaged in the task (distracted from the movies), and confirms that performance in the recognition task was not based on active working memory. Examination of the linguistic task verified a significant difference between the low and high attentional load conditions, thus validating the attentional load manipulation. The analysis revealed a significantly higher accuracy level [easy: 97.11% (4.68); hard: 84.49% (11.41); *t*_(23)_ = 8.09, *p* < 0.001] and a shorter RT [easy: 0.88 s (0.09); hard: 1.1 s (0.13); *t*_(23)_ = -19.85, *p* < 0.001] for the easy compared to the hard linguistic questions.

Finally, the influence of the attentional load manipulation (which occurred during encoding) on the ability to recognize a stimulus from the encoded movies (which was assessed during recognition) was examined. Although the low and high attentional load conditions did not significantly differ in terms of accuracy level [low load: 74.16% (12.8); high load: 71.87% (11.55); *F*_(1,23)_ = 0.6, *p* = 0.4], there was a significant effect in RT. Thus, participants recognized stimuli from movies encoded during the low load condition faster than the high load condition [low load: 1.76 s (0.3); high load: 1.82 s (0.34); *F*_(1,23)_ = 4.41, *p* = 0.047].

### fMRI Results

#### Inter-SC Analysis

**FIGURE 2:**
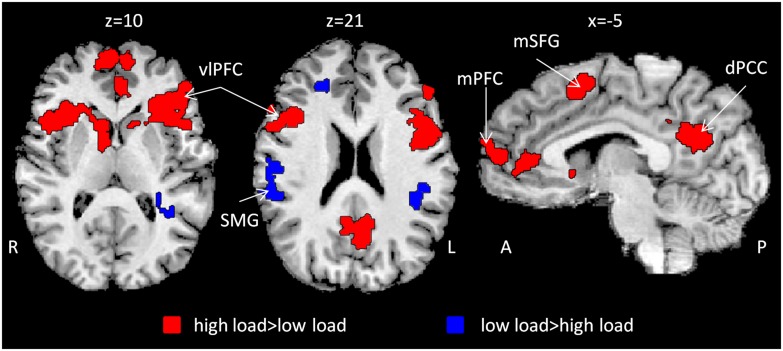
**Inter-SC as a function of attentional load.** Whole brain t-map depicting changes in inter-SC level as a function of attentional load (red and blue). Inter-SC increased during high attentional load in prefrontal regions and the dPCC (red). *n* = 22, *p* < 0.05, FDR corrected, cluster size > 50 × 3^3^. Abbreviations: R: right; L: left; A: anterior; P: posterior; vlPFC: ventrolateral prefrontal cortex; SMG: supramarginal gyrus; mSFG: medial superior frontal gyrus; mPFC: medial prefrontal cortex; dPCC: dorsal posterior cingulate cortex; inter-SC: intersubject correlation.

**FIGURE 3:**
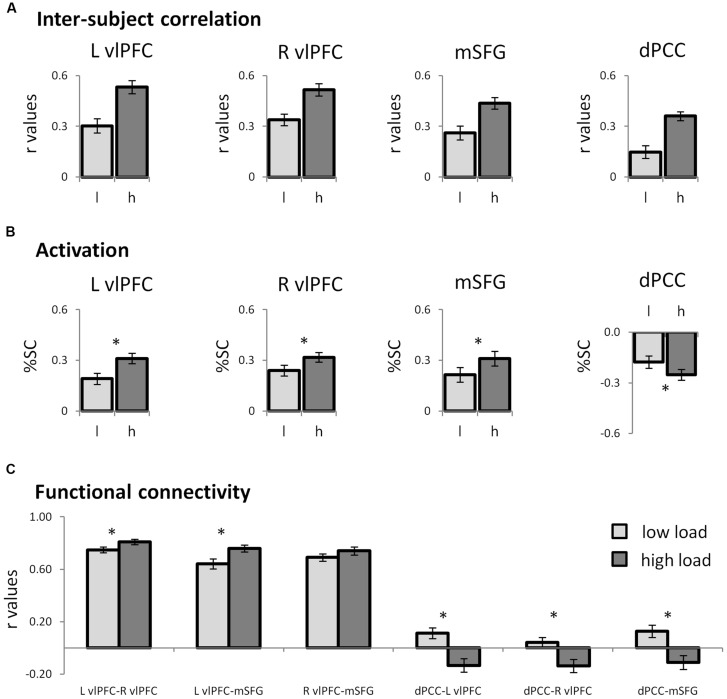
**Neural patterns of regions of interest. (A)** Bar graphs representing the inter-SC levels of the regions that emerged in the whole-brain inter-SC analysis, as depicted in **Figure 2**. **(B)** Bar graphs showing the results of the ROI activity analysis. The prefrontal regions were activated while the dPCC was deactivated at higher attentional load. In the prefrontal regions, the changes in inter-SC and activation were in the same direction, while in the dPCC they were in the opposite direction. **(C)** Functional connectivity between the ROIs. The prefrontal regions operated as a network, which during high load was more unified within itself and anti-correlated from the DMN hub, the dPCC. In all graphs error bars represent standard errors. Note that although statistical analyses were conducted on Fisher-transformed correlations, in all the graphs results are presented in the original correlation, for the reader’s convenience. Abbreviations: L: left; R: right; l: low load; h: high load; vlPFC: ventrolateral prefrontal cortex; dPCC: dorsal posterior cingulate cortex; SMG: supramarginal gyrus; inter-SC: intersubject correlation; %SC: percent signal change from baseline. ^∗^*p* < 0.05, Bonferroni corrected.

Correlation maps for low and high attentional load conditions were created (Supplementary Figure S1) and compared using a paired-sample *t*-test in order to depict regions which changed their response reliability as a function of attentional load. Higher response reliability was generally observed in the high compared to the low attentional load (**Table 1**). Specifically, responses were more synchronized in the high load condition in bilateral vlPFC, medial superior frontal gyrus (mSFG), mPFC, ventral precuneus/dorsal posterior cingulate cortex (dPCC) and bilateral caudate (**Figure 2**, red; **Figure 3A**). Only a few regions demonstrated the opposite effect, including the supramarginal gyrus (SMG) bilaterally, right dorsal precuneus and anterior middle frontal gyrus (**Figure 2**, blue). The unthresholded correlation maps and t-map can be viewed in Neurovault (Gorgolewski et al., 2015)^1^.

**Table 1:** Inter-SC results.

Region	Inter-SC effect	Talairach coordinates			Number of voxels	*t*_(21)_	*p*-value	Activation effect
		*X*	*Y*	*Z*				
L Supramarginal gyrus	Low > High	-42	-37	22	2414	7.81	<0.000001	Low ∼ = High, %SC > Base
L Ventrolateral prefrontal cortex	High > Low	-30	20	4	21530	-9.2	<0.000001	High > Low, %SC > Base
Medial prefrontal cortex	High > Low	-9	56	13	5325	-6.91	0.000001	Low ∼ = High, %SC < Base
Dorsal posterior Cingulate Cortex/Ventral precuneus	High > Low	0	-52	31	4532	-6.82	0.000001	Low > High, %SC < Base
Medial superior frontal gyrus	High > Low	3	14	49	4204	-7.06	0.000001	High > Low, %SC > Base
R Dorsal precuneus	Low > High	9	-76	28	1593	5.22	0.00003	Low ∼ = High, %SC ∼ = Base
L Anterior middle frontal gyrus	Low > High	24	38	29	2550	6.05	0.000005	High > Low, %SC < Base
R Ventrolateral prefrontal cortex	High > Low	39	17	19	14860	-8.54	<0.000001	High > Low, %SC > Base
R Supramarginal gyrus	Low > High	54	-34	19	2353	7.77	<0.000001	Low > High, %SC > Base

*Regions demonstrating change in inter-SC level as a function of attentional loading. n = 22, p < 0.05, FDR corrected, cluster size > 50 × 3^3^ voxels. Abbreviation: Inter-SC: inter-subject correlation; L: left; R: right; Low: low attentional load; High: high attentional load; %SC: percent signal change from baseline; Base: baseline; ∼ = : conditions are not significantly different.*

#### Characterizing Signal Level via Activation Analysis

In the regions which demonstrated inter-SC changes, activation levels were calculated and used to characterize the regions as either TPN or DMN. In all regions except the right dorsal precuneus, signal level was significantly different from zero, indicating that the average amplitude during the conditions was different from the amplitude during fixation (**Table 1**). Congruent to the inter-SC results, the bilateral vlPFC and the mSFG were more activated during the high than the low attentional load condition (**Figure 3B**). As the %SC in these regions was higher than baseline, they were defined as TPN regions. The ventral precuneus/dPCC was more deactivated in the high than the low attentional load condition, and was thus classified as DMN. These results suggest that in the TPN, the inter-SC and activity level changed in the same direction, while in the DMN they changed in the opposite direction. The other regions that demonstrated significant inter-SC patterns showed changes in activation level that did not match either network. The three TPN and one DMN regions were defined as ROIs for subsequent analyses.

#### Functional Connectivity Analysis

Functional connectivity was used to assess the relationship within and between functional networks as related to attentional load. Within the TPN regions, correlation between each pair of prefrontal regions was positive and increased as the task became harder (**Figure 3C**; **Table 2**). Regarding the relationship between the TPN and DMN, the high attentional load condition was characterized by negative correlations between each of the prefrontal regions and the dPCC. These findings indicate that the prefrontal regions of the TPN were more coherent and more anti-correlated to the dPCC as attentional load increased.

**Table 2:** Functional connectivity between pairs ROIs.

	low load, *r*_(22)_	high load, *r*_(22)_	*t*_(21)_	*p*-value
L vlPFC-R vlPFC	0.75	0.81	-3.25	0.02
L vlPFC-mSFG	0.64	0.76	-4.7	0.0006
R vlPFC-mSFG	0.69	0.74	-2.89	0.05
L vlPFC-dPCC	0.11	-0.13	6.42	<0.00001
R vlPFC-dPCC	0.04	-0.14	4.03	0.003
mSFG-dPCC	0.13	-0.11	5.01	0.0004

*For each pair of ROIs, FC was examined using a paired sample t-test. Correction for multiple comparisons was done using the Bonferroni method (i.e., multiplying the p-value by 6).*

#### Functional Significance of Inter-SC Patterns

**FIGURE 4:**
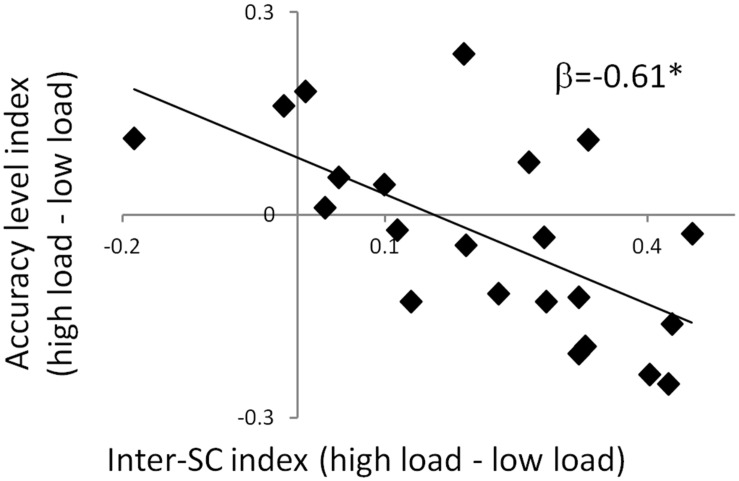
**Predicting accuracy level via inter-SC in the dPCC.** A scatter plot of the regression model that predicts interference to memory using inter-SC of the dPCC. The significant negative regression coefficient indicates that increase in inter-SC during the high attention condition was associated with greater memory impairment in that condition. This suggests that modulation of inter-SC level of the dPCC by attention predicts memory interference. ^∗^*p* < 0.05. Note that although statistical analyses were conducted on Fisher-transformed correlations, the results are presented in the original correlation, for the reader’s convenience.

To clarify the functional significance of the inter-SC patterns, we examined their ability to predict memory strength. This was done using a step-wise multiple regression analysis with the inter-SC index of the TPN and DMN ROIs as the predicting variables. The model that predicted the accuracy level index of the mnemonic task contained only the inter-SC index of the dPCC, which explained 37.5% of the variance [*R*^2^ = 0.375, Adj-*R*^2^ = 0.344, *F*_(1,20)_ = 12, *p* = 0.002; β = -0.612; **Figure 4**]. This means that a larger increase in inter-SC level of the dPCC during the high attentional load predicted lower memory strength in that condition. In order to clarify whether this relationship was specific to inter-SC patterns, a parallel model was examined with the activation indices as the predicting variables and one with the FC indices as predicting variables. These models were non-significant, suggesting a specific relationship of memory to inter-SC. Finally, the model that predicted RT index of the mnemonic task was non-significant for either inter-SC, activation or FC indices.

## Discussion

The current study portrayed how attention modulates the interplay between processing and memory of dynamic stimuli by utilizing movies and analyzing inter-SC. We found that high attentional load was associated with both lower memory strength and higher synchronization of neural signal across participants in lateral and medial prefrontal regions and dPCC. The experimental design also allowed a unique opportunity to further characterize activity level in each region as well as their functional coupling, using activation and FC methods, respectively. We found that in the prefrontal TPN regions, both inter-SC and activation increased at higher attentional load, and FC within the network was higher. In contrast, in the dPCC, higher attentional load was accompanied by greater inter-SC, greater deactivation and greater anti-correlation with the TPN regions. Critically, only the inter-SC level of the dPCC predicted memory strength.

In terms of behavior, responses to the easy linguistic task were faster and more accurate than the hard linguistic task, confirming two levels of attentional load during encoding. Importantly, encoding during low attentional load was associated with shorter response latency for the mnemonic questions that followed. Since this measure is considered an indication for memory strength (Stanners et al., 1969; Sternberg, 1969), this result implies that a stronger memory was created when interference during encoding was less extensive.

The inter-SC method is specifically designed to depict neural dynamics evoked by a continuous stimulus (Hasson et al., 2015). We found that high attentional load was associated with greater inter-SC in prefrontal regions and in the dPCC (**Figure 2**), which are known to accumulate information across long timescales (Lerner et al., 2011). These findings support Hasson et al.’s (2015) framework, where attention modulates the interplay between processing and memory in high order regions. Increase inter-SC indicates greater coupling between the region’s activity and the ongoing stimulus (van Kesteren et al., 2010). Thus, activity in these regions was more stimulus-bound to the combination of the movie and the secondary task at higher attentional load. However, greater inter-SC level of the dPCC during encoding with high attentional load predicted lower memory strength of the movie, as indicated by accuracy level during the recognition task (**Figure 4**). Given the known involvement of the PCC in mnemonic operations (Svoboda et al., 2006; Vann et al., 2009; Shapira-Lichter et al., 2013), we suggest that in the high attentional load condition, the PCC was more attuned to the secondary linguistic task at the expense of the movie. A higher whole-brain inter-SC score has been previously associated with greater attentional control (Campbell et al., 2015), and here we focus this finding to a particular region. Furthermore, while the inter-SC level of regions such as the parahippocampal gyrus and superior temporal gyrus predicts subsequent memory (Hasson et al., 2008a), our data suggest that the inter-SC level of the dPCC predicts interference to memory. The idea that the inter-SC level of the dPCC is a marker for interference is in line with previous findings, including that expert mediators who enjoy higher levels of attention and concentration show improved regulation of PCC function, and that the PCC is the target for neurofeedback training that aims, among other things, to reduce distractibility (Brewer and Garrison, 2014).

Combining the inter-SC and activation analysis allowed us to further characterize the regions which presented significant inter-SC patterns. We found that in the bilateral vlPFC and mSFG, increases in inter-SC were accompanied by higher activations (**Figures 3A,B**) as anticipated from TPN regions. These activation results are in agreement with previous studies that utilized still stimuli and measured activity, which showed that dividing attention during encoding recruited lateral and medial prefrontal regions (Uncapher and Rugg, 2005, 2008; Johnson and Zatorre, 2006; Rissman et al., 2009). The prefrontal regions are known to be important in cognitive control (Duncan and Owen, 2000; Duncan, 2013). Thus, the two analytic methods complemented each other, with the inter-SC method suggesting the potential processes underlying the activation changes. The activation results indicated recruitment of prefrontal control regions in the face of high attentional load, and inter-SC patterns suggested that this was accomplished via greater attunement to the simultaneous stimuli. A different relationship between inter-SC and activation was observed in the dPCC, which was more synchronized and more deactivated when encoding was conducted with high attentional load (**Figures 3A,B**). This activation pattern is expected since the PCC is part of the DMN, which is known to be deactivated as load increases (Raichle et al., 2001). As mentioned above, the inter-SC level was a marker for interference during encoding.

Functional connectivity analysis revealed that as attentional load increased, the prefrontal section of the TPN network became coherent within itself and negatively correlated with the dPCC (**Table 2**; **Figure 3C**). This is in line with finding that the anti-correlation between the DMN and TPN increases at higher loading (Newton et al., 2011). This anti-correlation was previously shown to represent a modulation that the PCC imposes on the prefrontal regions (Uddin et al., 2009). Therefore, our findings that the PCC is both a marker for interference in memory processing and it is anti-correlated with the TPN, combined with the findings of Uddin et al. (2009), may suggest that the attunement of the PCC to the secondary task necessitated recruitment of prefrontal control mechanisms.

Together, our results show that attentional load modulated the function of regions known to accumulate information over long timescales when facing continuous real-life stimuli (Lerner et al., 2011). The modulation of attention manifested foremost as an increase in synchronization of neural signal. The pivotal area for the impact of this attunement was the dPCC, which had to juggle between its involvement in episodic memory (Svoboda et al., 2006; Vann et al., 2009; Shapira-Lichter et al., 2013) and dealing with the interference that disrupted the ongoing mnemonic task (Leech and Sharp, 2014). As load increased and the secondary task became more demanding, it tracked the interfering stimuli instead of the movie, thus weakening memory. The dPCC also displayed anti-correlation with the prefrontal regions at higher attentional load, possibly influencing their function (Uddin et al., 2009) and causing recruitment of these prefrontal control mechanisms. Future studies may address the question of how a parametric rather than dichotomous increase in attentional load would affect neural patterns, and what determines the ability of these systems to cope with increasing attentional demand.

## Author Contributions

NO: Substantial contributions to the conception and design of the work, acquisition, analysis, and interpretation of data and writing the work. IS-L: substantial contributions to the conception and design of the work, analysis, and interpretation of data and revising the work critically. NO and IS-L share first authorship equally. YL and RT: substantial contributions to the analysis and interpretation of the data and revising the work critically. TH, NG, and ELA: substantial contributions to the conception of the work and revising the work critically. All authors: final approval of the version to be published and agreement to be accountable for all aspects of the work.

## Conflict of Interest Statement

The authors declare that the research was conducted in the absence of any commercial or financial relationships that could be construed as a potential conflict of interest.

## Footnotes

1 http://neurovault.org/collections/1871/
