# Supplementary material for: How Attention Modulates Encoding of Dynamic Stimuli
Source: Front Hum Neurosci. 2016 Oct 21;10:507. doi: 10.3389/fnhum.2016.00507 (PMC5073125; doi:10.3389/fnhum.2016.00507)
Supplement: Supplementary file 1 [file Data_Sheet_1.docx]

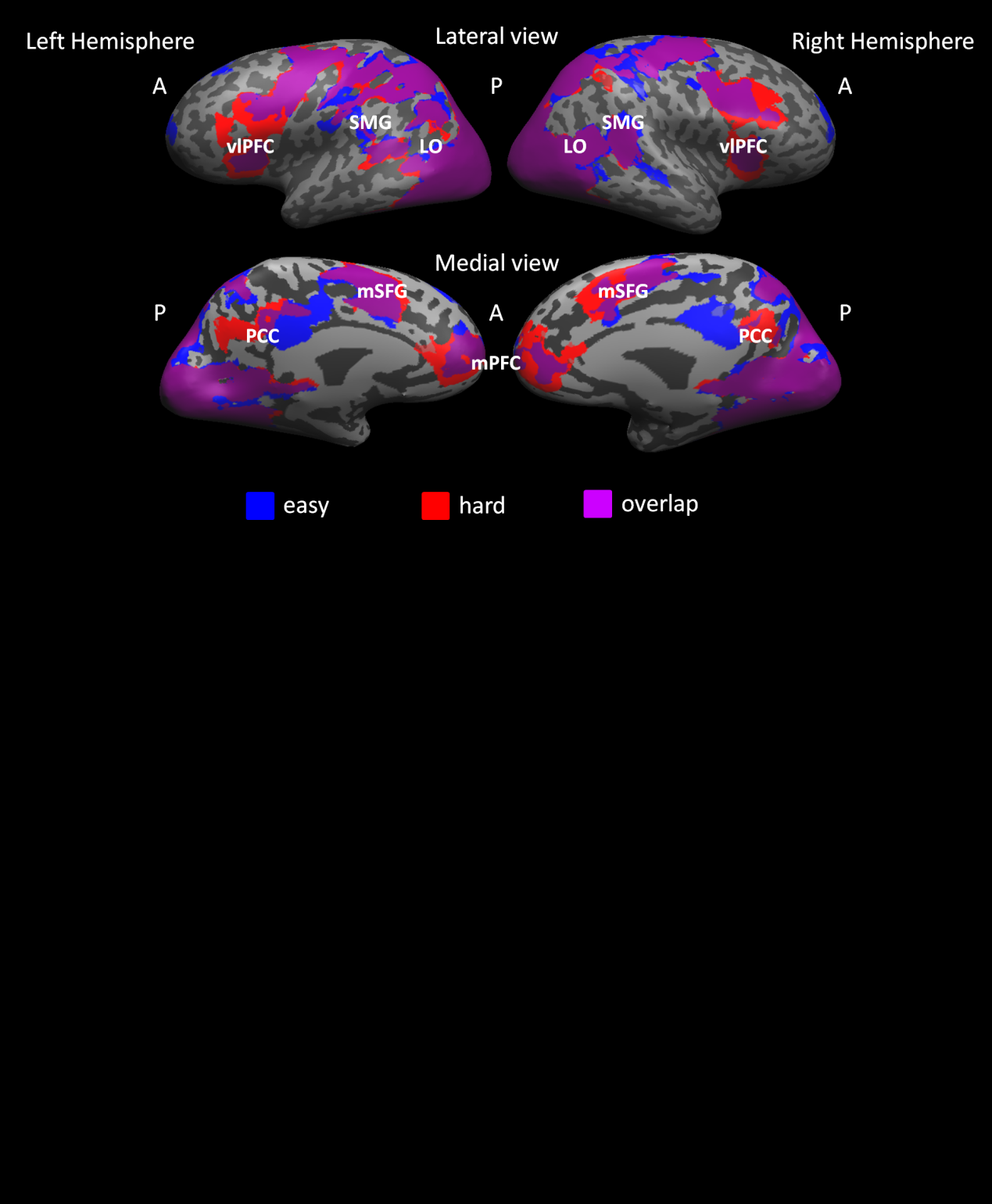


**Figure S1. Correlation maps of low and high load conditions.**

Maps of response reliability across participants as a function of attentional load, presented in lateral and medial views. Pearson correlation coefficients were calculated on a voxel-by-voxel basis, and maps for the low load (blue) and the high load condition (red) and their overlap (magenta) were created. The maps illustrate the similarity between reliable responses evoked in different conditions. Reliability of response was first computed in each specific condition (e.g., low load first movie) for each participant and movie, and only later was averaged – first across movies and then across participants (see Material and methods). The threshold is r > 0.15, cluster size > 50 x 3^3^. Abbreviation: A: anterior; P: posterior; RH: right hemisphere; LH: left hemisphere; vlPFC: ventrolateral prefrontal cortex; SMG: supramarginal gyrus; LO: lateral occipital; mSFG: medial superior frontal gyrus; vmPFC: medial prefrontal gyrus.
